# Supporting Caregivers in Clinical Practice: Findings From a Pilot Randomized Controlled Trial

**DOI:** 10.1093/geroni/igaf122.180

**Published:** 2025-12-31

**Authors:** Ronald Adelman

**Affiliations:** Weill Cornell Medicine, New York, New York, United States

## Abstract

Caregivers commonly accompany older adults to their routine medical visits, yet they are not systematically supported in clinical practice. Caregivers report feeling ill-prepared for their role and receive little guidance from healthcare professionals. The clinical component of our Caregiver Program aims to address these issues by launching a comprehensive approach to family-centered care that incorporates caregiver assessment, training, and support. As a first step in this initiative, our multidisciplinary team developed Collaborative Healthcare Encounters with Caregivers (CHEC), a checklist-based intervention to help caregivers identify and communicate their concerns to healthcare providers. We evaluated the feasibility, acceptability, and preliminary efficacy of CHEC in a randomized controlled pilot trial of N = 52 patient-caregiver dyads recruited from our geriatric outpatient practice. Visits were audio-recorded and analyzed using a standardized coding procedure. Post-visit questionnaires assessed caregivers’ perceptions of the checklist, efficacy in primary care interactions and knowledge of relevant resources. CHEC caregivers reported that the checklist was easy to complete (89.3%) and useful in helping the medical team to recognize their concerns (78.6%). Analyses of audio-recordings found that CHEC visits allocated a greater proportion of the visit time to discussing caregivers’ concerns about the patient. As compared to usual care participants, CHEC caregivers reported higher efficacy in primary care interactions and greater knowledge of relevant resources. These findings suggest that CHEC is feasible to administer and may promote greater involvement of caregivers in patient-centered discussions. Results lay the groundwork for future activities aimed at providing comprehensive education and support to caregivers throughout our clinical programs.

